# Determinants of treatment completion among rural smear positive pulmonary tuberculosis patients: a cross-sectional survey conducted in south-western Uganda

**DOI:** 10.1186/s40249-017-0313-3

**Published:** 2017-07-04

**Authors:** Edgar Mugema Mulogo, Christopher Nahabwe, Fred Bagenda, Vincent Batwala

**Affiliations:** 1 Department of Community Health, Mbarara University of Science and Technology, PO Box 1410, Mbarara, Uganda; 2 Rwampara Health Sub-District, PO Box 1, Mbarara, Uganda

**Keywords:** Tuberculosis, Treatment completion, Determinants, Rural Uganda

## Abstract

**Background:**

Treatment completion among tuberculosis patients remains low across various regions of Uganda, despite implementation of directly observed treatment short course. This study evaluated the determinants of treatment completion in a rural health sub-district of south western Uganda.

**Methods:**

In April 2012, health facility records were reviewed to identify tuberculosis patients who had been initiated on treatment between June 2008 and May 2011, in Rwampara Health Sub-District, south-western Uganda. Out of the 162 patients identified, 128 (79%) were traced and subsequently interviewed during a survey conducted in June 2012. Eleven (6.8%) of the 162 patients died, while 23 (14.2%) could not be traced. A review of records showed that 17 of those that could not be traced completed treatment while the other six did not have definitive records.

**Results:**

Treatment completion among the 128 patients interviewed was 89.8%. Pre-treatment counselling (a*OR* = 24.3, 95% *CI*: 1.4–26.6, *P* = 0.03), counselling at the time of submission of sputum during follow up (a*OR* = 6.8, 95% *CI*: 1.4–33.7, *P* = 0.02), and refill of drugs on the exact appointment date (a*OR* = 13.4, 95% *CI*: 1.9–93.0, *P* = 0.01), were independently associated with treatment completion.

**Conclusions:**

The level of treatment completion was higher than the national average, with service- related determinants identified as being critical for ensuring treatment completion. These data provide further evidence for the need to provide ongoing counselling support to tuberculosis patients. Enhancing the opportunities for counselling of tuberculosis patients should therefore be rigorously promoted as an approach to increase treatment completion in rural settings.

**Electronic supplementary material:**

The online version of this article (doi:10.1186/s40249-017-0313-3) contains supplementary material, which is available to authorized users.

## Multilingual abstracts

Please see Additional file 1 for translations of the abstract into the five official working languages of the United Nations.

## Background

The tuberculosis (TB) burden and infection rates have remained high despite internationally recommended strategies to contain the disease. In 2014, an estimated 9.6 million new TB cases were reported worldwide: 5.4 million among men, 3.2 million among women and 1.0 million among children [1]. The African region accounts for 28% of the reported cases [2].

The currently recommended treatment for new cases of drug-susceptible TB is a 6-month regimen of four first-line drugs: isoniazid, rifampicin, ethambutol and pyrazinamide [1]. Although TB is curable the emergence of drug-resistant strains of the causative Mycobacterium genus is a global threat to its control [3]. Therefore, failure to adhere to the prescribed regimen measured as “treatment completion”, is cited as one of the greatest problems facing the current control strategies [4]. “Treatment completion” together with “treatment success” are some of the indicators to measure performance of national TB control programs [1, 5]. An earlier study suggested that increasing patient-centeredness of interactions between providers and clients promotes TB treatment adherence [6], yet as a component of pillar 1 of the End TB Strategy, the World Health Organization (WHO) recommends the need for all TB patients to receive educational, emotional, and economic support to enable them to complete treatment [6, 7]. A study in South Africa reported that adjustments in access to the health-care system enhanced TB treatment completion [8]. The WHO has set treatment success target of ≥ 90% by the year 2025 [7].

Uganda remains a high burden country, with a prevalence rate of 161 TB cases per 100 000 people [9–11]. Nationally, Uganda’s TB and Leprosy Prevention and Control Program was not able to achieve targets for indicators related to treatment success and cure rates in the year 2014/15. This has been attributed to low adherence to treatment as evidenced by the low achievement (68% of TB patients) of directly observed treatment, short course (DOTS) [12]. Other challenges faced by the Uganda’s TB program relate to inadequate systems for TB case detection, adherence monitoring and testing relapses for drug sensitivity [12].

The Rwampara Health Sub-District (HSD) is located in the Mbarara District of south western Uganda. It has a population of 152 600, which is served by five TB treatment centers (including four health centers at sub-county level and one at county level). In 2011, the HSD reported a treatment success of 55% [13]. This was below that reported at the national level which ranged from 65 to 73% among new cases, from 2009 to 2011 [9, 14]. In response to this, the present study evaluated the determinants of TB treatment completion in Rwampara HSD for the period from 2009 to 2011.

## Methods

### Study setting and sample size

In April 2012, the study team reviewed health facility records of all TB patients who had been initiated on treatment between June 2008 and May 2011, in Rwampara HSD. A survey was then conducted among the patients identified from the records in June 2012.

The study participants were selected from among those receiving TB treatment in the HSD regardless of the TB management strategy deployed, i.e. community-based (CB) versus health facility-based (HF) DOTS. The participants included both new and retreatment smear positive pulmonary TB patients. Tuberculosis patient registers were obtained from the five treatment centres and the study participants were identified. The treatment centre registers had a total of 162 patients who had started treatment between June 2008 and May 2011, and were expected to have completed treatment by the commencement of the survey in 2012. All the identified patients (162) were included in the survey as potential participants. The participants were traced in the community with the guidance of community health workers and village leaders who acted as field guides.

### Data Collection

#### Questionnaire design and administration

A standardized interviewer administered questionnaire was used during the patient survey to collect information on socio-demographic data; knowledge on TB treatment; treatment practices by patients, and community and health workers; adherence to TB medication; and health facility-based factors such as drug availability, health worker availability, and waiting time. Medical records were reviewed for information on the demographics, counselling, HIV testing, cadre of health workers available to treat patients, treatment strategy, medical commodities available, and treatment completion pertaining to patients in order to determine whether they were transferred, could not be traced, died, or had completed treatment successfully.

The questionnaire was pretested at a treatment centre on patients who were on treatment in order to identify any problems associated with its interpretation. Then findings from the pre-test were used to improve the wording of the questions, their sequence, as well as the length of the questionnaire.

#### Definitions of variables

The variables were defined as follows:

Treatment completion was defined as “a TB patient who completed treatment without evidence of failure but there was no record to show that sputum smear or culture results in the last month of treatment and on at least one previous occasion were negative, either because they were not done or because results were not available” [4, 5]. Patients were classified as having completed treatment if they had swallowed their medication for the prescribed period.

Adherent patients were those who swallowed the drugs as prescribed daily for 6 months consistently (the recommended period for TB regimen), while non-adherent were patients who had interruptions in taking TB medication during the 6 month period. Patients were classified as adherent if they swallowed 90% of the total medication they were given.

Access to the health unit providing TB services was defined as living within a physical distance of five kilometres (≤5 km) from a health facility.

Waiting time was measured as the period a patient spends at a treatment centre from the time of arrival to when a TB-related service is provided. Waiting time was measured in hours and compared against the Government of Uganda recommendation of 1 h [15, 16].

### Data analysis

The analysis was done using STATA version 12 software [17]. Two-sided chi-square tests for association were computed to detect differences between categorical variables such as sex, alcohol consumption, drug side effects, presence of treatment supporter, family support, drug availability, and waiting time. The means of continuous variables were compared using *t*-tests. The strength of association was interpreted based on a sliding scale of *P*-values.

In order to investigate the relationship between the outcome variable (treatment completion) and other variables, logistic regression models were run. The model building strategy was not only limited to significant variables from the bivariate analysis, but also included independent variables that were considered to have a clinical and social significance for the outcome [18, 19]. These independent variables were: pre-treatment counselling, health worker availability, counselling at the time of submission of follow-up sputum, timely medicine refills, adherence to treatment, waiting time at health facility, and whether the patient had been tested for HIV. The demographic variables were not included in the models because the two groups of patients (completed and did not complete treatment) were similar with regard to these characteristics.

The evaluation of the determinants of treatment completion was based on 128 participants due to missing information on several variables for those who had either died or been lost to follow-up.

## Results

### Tracing of study participants

**Fig. 1:**
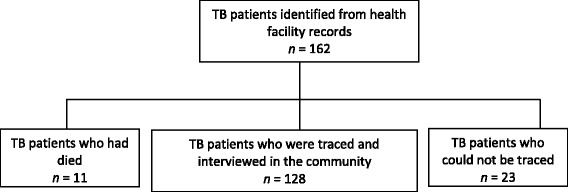
Study profile

Out of the 162 patients, 128 (79%) were traced and subsequently interviewed. Eleven (6.8%) of the 162 patients died while 23 (14.2%) could not be traced. A flow diagram of the participants is shown in Fig. 1.

A review of records showed that 17 of those who were not traced completed treatment, while the other six did not have definitive records.

### Patient sociodemographic characteristics

A record review of those alive showed that 115 (89.8%) had completed treatment. The sociodemographic characteristics of these patients are shown in Table 1.

**Table 1:** Patient sociodemographic characteristics

Characteristic		Treatment completion (*N* = 128)		*P*-value
		Yes (115) *n* (%)	No (13) *n* (%)	
Sex	Male	33 (28.7)	3 (23.1)	1.00^λ^
	Female	82 (71.3)	10 (76.9)	
Mean age (years) (SD)		43.3 (13.5)	41.7 (11.2)	0.68^¥^
Age range (years)		18–84	26–70	Not calculated
Marital status	Ever married	102 (88.7)	11 (84.6)	0.65^λ^
	Single	13 (11.3)	2 (15.4)	
Education	Formal	89 (77.4)	9 (69.2)	0.50^λ^
	No formal	26 (22.6)	4 (30.8)	
Having children	Yes	103 (89.6)	10 (76.9)	0.18^λ^
Monthly median income (IQR)		$8.8 ($2.9–$17.6)	$10.3 ($2.9–23.5)	-

IQR = interquartile range, *SD* = standard deviation, $ = US dollar, λ = Fisher’s exact Test, ¥ = *t* test

The majority of the patients were: female (71.3%), ever married (88.7%), and attained a level of formal education; the minimum level in this case was primary education (77.4%). Eighty-eight percent (88%) of the participants had children. The mean age in years was 43.3 for participants who completed treatment and 41.7 years for those who did not complete treatment. Their age ranges in years were 18–84 and 26–74, respectively. The two groups of study participants (completed and did not complete treatment) did not differ on all the characteristics reported in Table 1.

### Association between patient medical and other characteristics, and treatment completion

Table 2 compares the medical and other characteristics of patients, by treatment completion.

**Table 2:** Association between medical and other characteristics, and treatment completion among the 128 Ugandan TB patients in a bivariate analysis

Characteristic		Treatment completion (*N* = 128)		OR (95% *CI*)	*P*-value
		Yes (115) *n* (%)	No (13) *n* (%)		
Patient capable of walking at initiation of treatment	Yes	104 (90.4)	12 (92.3)	0.8 (1.0–6.6)	0.83
Distance > 5 km	Yes	57 (49.6)	7 (53 .9)	0.8 (0.3–2.7)	0.77
Had side effects	Yes	35 (30.4)	7 (53.9)	0.4 (0.1–1.2)	0.10
Adhered to treatment	Yes	90 (78.0)	7 (53.9)	0.3 (0.1–1.1)	0.06
Have children	Yes	103 (89.6)	10 (76.9)	2.6 (0.6–10.7)	0.19
Disclosed condition to spouse	Yes	81 (70.4)	9 (69.2)	1.3 (0.4–4.4)	0.62
Alcohol drinking	Never	83 (72.2)	4 (30.8)	5.8 (1.7–20.2)	*0.04* ^a^*

*SD* = standard deviation

^a^ Fisher’s exact test, *significant at *p* = 0.05

Patients who had never consumed alcohol (odds ratio, *OR* = 5.8, 95% confidence interval, *CI*: 1.7–20.2) were more likely to complete TB treatment. A larger proportion (54%) of the patients who did not complete treatment experienced side effects, however, this result was not statistically significant (*OR* = 0.4, 95% *CI*: 0.1–1.2).

### Association between service-related factors and treatment completion

Information on service-related factors for the patients was obtained from the records of the treatment centres. A comparison of these factors by treatment completion is shown in Table 3.

**Table 3:** Association between service-related factors and treatment completion among the 128 Ugandan TB patients in a bivariate analysis

Factor		Treatment completion		*OR* (95% *CI*)	*P*-value
		Yes (115)	No (13)		
Treatment strategy	CB-DOTS	100 (87.0)	10 (76.9)		
	HF-DOTS	15 (13.0)	3 (23.1)	2.0 (0.5–8.1)	0.32
Treatment category	New	98 (85.2)	11 (84.6)		
	Retreatment	17 (14.8)	2 (15.4)	1.0 (0.2–5.1)	0.95
Pre-treatment counselling	Yes	111 (96.5)	9 (69.2)	12.3 (2.6–57.7)	*0.01* ^a^*
Counselling at the time of submission of follow-up sputum	Yes	90 (78.3)	5 (38.5)	5.8 (1.7–19.2)	*0.04* ^a^*
Was tested for HIV	Yes	107 (93.0)	11 (84.6)	2.4 (0.5–12.9)	0.30
Waiting time > 1 h	Yes	25 (21.7)	5 (38.5)	0.4 (0.1–1.5)	0.19
Health worker available	Yes	112 (97.4)	11 (84.6)	6.8 (1.0–45.1)	*0.05* ^a^*
Drugs available always	Yes	98 (85.2)	11 (84.6)	1.0 (0.2–5.2)	0.95
Timely refill of drugs	Yes	74 (64.4)	2 (15.4)	9.9 (2.1–47.0)	*0.04* ^a^*
Complete records at facility	Yes	38 (33.0)	2 (15.4)	2.7 (0.6–12.9)	0.20

^a^ Fisher’s exact test, *significant at *p* = 0.05

Patients who were counselled before initiation of TB therapy were 12 times more likely to complete their treatment (*OR* = 12.3, 95% *CI*: 2.6–57.7). Counselling at the time of submission of sputum during follow-up (*OR* = 5.8, 95% *CI*: 1.7–19.2), availability of health workers at treatment centre (*OR* = 6.8, 95% *CI*: 1.0–45.1), and drug collection on exact appointment date, also known as timely refill (*OR* = 9.9, 95% *CI*: 2.1–47.0) were all significantly associated with treatment completion. Again, there was a very weak positive correlation between “pre-treatment counselling” and “counselling at the time of submission” of follow-up sputum samples during the course of the treatment (correlation coefficient “*r*” 0.143). However treatment strategy (CB-DOTS or HF-DOTs) and treatment category (new or retreatment case) were not significantly associated with treatment completion.

### Independent determinants of treatment completion

The results from the logistic regression analysis are presented in Table 4.

**Table 4:** Multivariable logistic regression showing the association between social, medical, and health service-related factors, and TB treatment completion

Variable	a*OR* (95% *CI*)	*P*-value
Pre-treatment counselling	24.3 (1.4–426.6)	0.03*
Health worker availability	1.9 (0.2–25.1)	0.61
Counselling at the time of submission of sputum during follow-up	6.8 (1.4–33.7)	0.02*
Timely refill of medicine	13.4 (1.9–93.0)	0.01*
Disclosed condition to spouse	1.3 (0.3–6.9)	0.72
Had side effects	0.5 (0.1–2.8)	0.45
Adhered to treatment	0.2 (0.1–1.3)	0.84
Waiting time > 1 h	1.4 (0.2–8.4)	0.69
Was tested for HIV	1.2 (0.1–18.1)	0.89

*Significant at *p* = 0.05

Pre-treatment counselling (adjusted *OR* = 24.3, 95% *CI*: 1.4–426.6, *P* = 0.03), counselling at the time of submission of sputum follow-up (a*OR* = 6.8, 95% *CI*: 1.4–33.7, *P* = 0.02), and refill of drugs on exact appointment date (a*OR* = 13.4 95% *CI*: 1.9–93.0, *P* = 0.01) were all found to be independently associated with treatment completion.

## Discussion

The findings show that the major determinants for treatment completion in this rural context were pre-treatment counselling, counselling at the time of the follow-up sputum submission visit, and timely drug refills. Consistent with studies conducted elsewhere counselling of TB patients impacts on treatment completion and better communication improves adherence [20–22]. A study conducted in Uganda reported that patients who are counselled more frequently tend to take their medication more consistently [23]. In the current study, the variation in the quality of counselling might have impacted the results. However, information on the quality of counselling was not collected and is an area for further investigation.

Good communication leads to understanding and therefore promotes observance of treatment requirements such as timely drug refills, resulting in improved treatment completion and cure [22]. On the other hand, the effects of non-dialogue counselling have been linked to non-adherence among TB patients [24]. Well-informed patients understand the importance of un-interrupted medication taking and patient knowledge about the dangers of not adhering to treatment is enhanced.

The treatment completion rate reported here is high when compared to the district and national rates at the time of the study [9, 13]. The lower district figures could be due to the reporting system failing to capture patient information in a number of cases. Patients tend to shift from one treatment centre to another without notifying the original treatment centre. This happens when drugs become unavailable at a given treatment centre and results in patients seeking medication at another centre. The original treatment centre then records these patients as defaulters and this is reflected in the district figures. Therefore, timely drug refill is a function of the health system factors (health workers’ knowledge and skills in managing TB patients, availability of drugs, etc) and the patient’s own responsibility of monitoring their personal drug stocks. In some studies, treatment supporters (who are often relatives) who monitor drug stocks and patients swallowing prescribed medicines have been used to counteract treatment interruption [25]. A study conducted in Nigeria reported that the attitude of health workers was associated with TB treatment interruption. It is suggested that the unfriendly attitude of health care providers may make patients feel threatened and unwelcomed leading to treatment interruption [26]. The attitude of health workers is also likely to affect TB patient participation in on-going counselling and therefore subsequently influence treatment completion.

In contrast to the findings of a review of studies conducted in Uganda and elsewhere, sex, education, distance to treatment centre, and treatment regimen, were not found to be associated with treatment completion [24, 27–30]. However, consistent with other study findings, TB treatment strategy was not associated with treatment completion [31]. Some patients choose the HF-DOTS strategy because they either live close or have transport to the health facility and trust health workers with the responsibility of their treatment. This group of patients is also likely to complete treatment as observed among patients using CB-DOTS, resulting in no significant difference between the two strategies.

Health worker availability and mean duration on treatment were not independently associated with treatment completion. This can mainly be attributed to the use of CB-DOTS treatment strategy by the majority of patients in this rural setting.

### Limitations

Although the study was confined to one HSD this study area is typical of other rural setting in terms of health infrastructure in Uganda. The study findings are therefore comparable across similar settings. We also note that this was a cross-sectional study and therefore we cannot define the temporal relationship between the independent variables and outcome. The direction of causality can therefore only be regarded as suggestive. The data collected on a number of independent variables were based on self-reports that are likely to be subject to social desirability bias. As a result there is a limit to which such responses can be considered accurate by foreknowledge of what, in the view of the respondent, would be a suitable response. However, the current findings do carry implications for health service managers, decision-makers, and health care providers in their consideration of the designing and implementing TB services.

## Conclusions

The findings demonstrate a high level of treatment completion among rural TB patients in the study setting, with service-related determinants identified as critical for ensuring treatment completion. These data provide further evidence for the need to provide ongoing counselling support to TB patients. Enhancing the opportunities for counselling of TB patients should therefore be rigorously promoted as an approach to increase treatment completion in rural settings.

In these contexts ongoing counselling builds the health provider patient relationship that is likely to result in treatment compliance and subsequently completion. However, for this to be realized, it is critical for TB programs in Uganda and elsewhere to ensure that timely refills are actualized in rural health facilities.

## Additional file

Additional file 1: Multilingual abstracts in the five official working languages of the United Nations. (PDF 585 kb)

## Abbreviations

a*OR*: Adjusted odds Ratio
CB-DOTS: Community-based DOTS
*CI*: Confidence interval
COHRE: International Clinical, Operational and Health Services Research
HF-DOTS: Health facility-based DOTS
HSD: Health sub district
JCRC: Joint Clinical Research Centre
p-value: Probability value
WHO: World Health Organization
