# Supplementary material for: Determinants of treatment completion among rural smear positive pulmonary tuberculosis patients: a cross-sectional survey conducted in south-western Uganda
Source: Infect Dis Poverty. 2017 Jul 4;6:104. doi: 10.1186/s40249-017-0313-3 (PMC5496174; doi:10.1186/s40249-017-0313-3)
Supplement: Additional file 1: — Multilingual abstracts in the five official working languages of the United Nations. (PDF 585 kb) [file 40249_2017_313_MOESM1_ESM.pdf]

Translation of the abstract into the five official working languages of the United Nations

العوامل المحددة لإكتمال عملية الشفاء بين مرضي المناطق الريفية الذين ثبت إصابتهم بالسل الرئوي بناءا على مسحة من الرئة: دراسة مقطعية أجريت في جنوب غرب أوغندا

إدغار ميوجيما مولوجو, كريستوفر نبوي, فريد باجيندا, فنسنت باتوالا

الملخص
الخلفية: يظل إكتمال الشفاء بين مرضي السل ذا نسبة منخفضة عبر العديد من المناطق في أوغندا وذلك على الرغم من تنفيذ دورة مباشرة قصيرة لملاحظة الشفاء من المرض وفي هذا الصدد فإن الدراسة الحالية تقيم العوامل المحددة لإكتمال عملية الشفاء في إحدى الوحدات الصحية الريفية الفرعية جنوب غرب أوغندا.
الأساليب: تم مراجعة سجلات المرافق الصحية في أبريل 2012 للتعرف على مرضي السل الدين تم البدء في علاجهم ما بين يونيه 2008 ومايو 2011 في وحدة روامبارا الصحية الفرعية في جنوب غرب أوغندا, تم الوصول لعدد 128 مريض من أصل 162 مريض (79٪) وتم عمل مقابلات معهم خلال عملية مسح ميداني واستقصاء أجريت عام 2012 , في حين انه قد مات أحد عشر مريضا (6.8٪) ولم يتم الوصول لعدد 23 مريض (14.2٪) من ال 162 مريض وبمراجعة التقارير تبين أن 17 مريض من الذين لم يتم الوصول اليهم كانوا بالفعل قد أكملوا علاجهم بينما الستة الآخرين لم يكن لهم سجلات محددة.
النتائج: كانت نسبة إكتمال الشفاء بين ال 128 مريض الذين تم مراجعتهم هي 89.8٪ وكانت إستشارة ما قبل العلاج (a*OR* = 24.3, 95% *CI*: 1.4 – 26.6, *P* = 0.03) والاستشارة عند تقديم عينة البصاق أثناء المتابعة (a*OR* = 6.8, 95% *CI*: 1.4 – 33.7, *P* = 0.02) وكذلك إعادة ملئ العلاج عند الموعد المحدد (a*OR* = 13.4, 95% *CI*: 1.9 – 93.0, *P* = 0.01) مرتبطة بشكل مستقل بإكتمال الشفاء من السل.
الاستنتاجات: كان مستوي إكتمال عملية الشفاء أعلي من المتوسط القومي وكانت بعض العوالم المحددة المتعلقة بتقديم الخدمة لها دور حاسم في إتمام الشفاء, وتقدم هذه البيانات مزيدا من الأدلة على مدي الحاجة لتوفير دعم الإستشارة المستمرة لمرضى السل وبالتالي فإن تعزيز فرص تقديم الإستشارة لمرضي السل يجب التنبيه عليه كمنهج لزيادة نسب إكتمال الشفاء في البيئات الريفية.

Translated from English version into Arabic by Mohamed Habib

涂片阳性肺结核患者治疗完成的决定因素：在乌干达西南部进行一项横断面调查

Edgar Mugema Mulogo, Christopher Nahabwe, Fred Bagenda, Vincent Batwala

摘要
引言：尽管实施了直接观察短期疗程，但在乌干达各地区结核病患者的治疗完成率仍很低。本研究评估了乌干达西南部一个农村卫生社区肺结核治疗完成的决定因素。
方法：2012 年 4 月，审查卫生设施记录以确定 2008 年 6 月-2011 年 5 月期间乌干达西南部的卢旺达拉健康小区接受治疗的结核病患者。在已确定 162 例患者中，追踪 128 例（79%），并在 2012 年 6 月进行随访。162 例患者中有 11 例（6.8%）死亡，23 例（14.2%）无法追踪。记录检查显示，其中 17 例无法追踪完成治疗，其余 6 例无确定记录。
结果：128 例接受随访的病例治疗完成率为 89.8%。治疗前咨询(a*OR* = 24.3, 95% *CI*: 1.4 – 26.6, *P* = 0.03)、在随访期间提交痰液时咨询(a*OR* = 6.8, 95% *CI*: 1.4 – 33.7, *P* = 0.02)、在确切的约定日期补充药物(a*OR* = 13.4, 95% *CI*: 1.9 – 93.0, *P* = 0.01)与治疗完成独立相关。
结论：治疗完成水平高于全国平均水平，服务相关决定因素是确保治疗完成的关键。这些数据为肺结核病患者提供持续的咨询支持提供进一步的证据。因此，应增加结核病患者咨询的机会，并将其作为提高农村肺结核治疗完成率的一种手段。

Translated from English version into Chinese by Lili Wang, edited by Pin Yang

Les facteurs liés à l'achèvement du traitement chez les patients atteints de tuberculose pulmonaire à frottis positif: une étude transversale menée dans le Sud-Ouest de l'Ouganda

Edgar Mugema Mulogo, Christopher Nahabwe, Fred Bagenda et Vincent Batwala

Résumé

Contexte: L'achèvement du traitement chez les patients atteints de tuberculose demeure faible dans diverses régions de l'Ouganda, malgré la mise en œuvre du traitement directement observé de brève durée. Cette étude a évalué les déterminants de l'achèvement du traitement dans un sous-district rural de santé du Sud-Ouest de l'Ouganda.

Méthodes: En avril 2012, les rapports des centres de santé ont été examinés afin d'identifier les patients atteints de tuberculose qui avaient été initiés au traitement entre Juin 2008 et Mai 2011, dans le Sous-District de Santé de Rwampara, au Sud-Ouest de l'Ouganda. Des 162 patients identifiés, 128 (79%) ont été répertoriés puis interrogés lors d'une étude menée en Juin 2012. Onze (6,8%) des 162 patients sont morts, tandis que 23 (14,2%) n'ont pas pu être retrouvés. Un examen des rapports a révélé que 17 de ceux qui ne pouvaient pas être retracés ont terminé leur traitement alors que les six autres n'ont pas de rapport définitif.

Résultats: L'achèvement du traitement parmi les 128 patients interrogés était de 89,8%. Le prétaitement (a*OR* = 24.3, 95% *CI*: 1.4 – 26.6, *P* = 0.03), la consultation au moment de la présentation de l'expectoration au cours de la période de suivi (a*OR* = 6.8, 95% *CI*: 1.4 – 33.7, *P* = 0.02), et le renouvellement des médicaments à la date fixée (a*OR* = 13.4, 95% *CI*: 1.9 – 93.0, *P* = 0.01), sont associés de façon indépendante à l'achèvement du traitement.

Conclusion: Le taux d'achèvement du traitement était plus élevé que la moyenne nationale, avec des facteurs liés à la prestation, identifiés comme étant d'importance majeure garantissant un traitement complet. Ces données fournissent une preuve supplémentaire de la nécessité d'offrir des consultations continues à des patients atteints de tuberculose. Le renforcement des possibilités de consultation de la tuberculose chez des patients doit donc être rigoureusement promu comme une approche visant à accroître l'achèvement du traitement en milieu rural.

Translated from English version into French by Ephrenm de Vera

Детерминанты завершения лечения среди сельских положительных мазок больных туберкулезом легких: кросс-секционные исследования, проведенного в юго-западной Уганде

Edgar Mugema Mulogo, Christopher Nahabwe, Fred Bagenda and Vincent Batwala

Реферат

Фон: Завершение лечения среди больных туберкулезом остается на низком уровне в различных регионах Уганды, несмотря на осуществление лечения под непосредственным наблюдением короткого курса. Это изучение оценило детерминанты завершения лечения в районных сельских врачебных пунктах в юго-западной Уганде.

Методы: В апреле 2012 года, учетные записи медицинского учреждения были проверены

для выявления больных туберкулезом, которые были начаты на лечении в период с июня 2008 года по май 2011 года в Rwampara Health Sub-District юго-западного Уганды. Из 162 пациентов были выявлены, что 128 (79%) были обнаружены и впоследствии опрошенный в ходе опроса, проведенного в июне 2012 года. Одиннадцать (6.8%) из 162 пациентов умерли, в то время как 23 (14.2%) не прослеживается. Анализ записей показал, что 17 из тех, что не может быть прослежены закончить лечение, а остальные шесть не имеют окончательные записи.
Результаты: Завершение лечения среди 128 опрошенных пациентов составляло 89,8%. Предварительная обработка консультирования (a*OR*= 24.3, 95% *CI*: 1.4 – 26.6, *P* = 0,03), консультации во время представления мокроты за последующие (a*OR*= 6.8, 95% *CI*: 1.4 – 33.7, *P* = 0,02), и пополнят запас лекарства на точную дату назначения (a*OR*= 13.4, 95% *CI:* 1.9 – 93.0, *P* = 0,01), были независимо связаны с обработкой завершения.
Заключение: Уровень завершения лечения был выше, чем национальный средний уровень, и также детерминанты, связанны с услугами были определены в качестве наиболее важных обеспечений завершения лечения. Эти данные подтверждают необходимость оказания постоянной консультационной поддержки для больных туберкулезом. Расширение возможностей для консультирования больных туберкулезом потом следует тщательно пропагандироваться как подход к повышению завершения лечения в сельской местности.

Translated from English version into Russian by Hao-Qi Zhang

Determinantes de la terminación del tratamiento entre los pacientes de tuberculosis pulmonar positiva de área rural: Encuesta transversal realizada en el suroeste de Uganda

Edgar Mugema Mulogo, Christopher Nahabwe, Fred Bagenda and Vincent Batwala

Abstracto

Trasfondo: La terminación del tratamiento entre pacientes de tuberculosis permanece baja en varias regiones de Uganda, a pesar de la implementación de un tratamiento de corta duración, que es directamente observado. Este estudio evaluó cuáles son los determinantes para la terminación del tratamiento un sub-distrito de salud rural del suroeste de Uganda.
Métodos: En abril de 2012, los récords de las facilidades de salud fueron revisadas para identificar pacientes de tuberculosis que iniciaron tratamiento entre junio de 2008 y mayo de 2011, en el sub-distrito de Rwampara en el suroeste de Uganda. De los 162 pacientes identificados, 128 (79%) fueron rastreados y subsiguientemente entrevistados durante una entrevista realizada en junio de 2012. Once (6.8%) de los 162 pacientes murieron, mientras que 23 (14.2%) no pudieron ser rastreados. Una revisión de los récords mostró que 17 de esos que no pudieron ser rastreados completaron el tratamiento mientras que los otros seis no tenían récords definitivos.
Resultados: La terminación del tratamiento entre los 128 pacientes entrevistados fue de 89.8%. Asesoramiento previo al tratamiento (a*OR* = 24.3, IC del 95%: 1.4 – 26.6, *P* = 0,03), asesoramiento al momento de la presentación del esputo durante el seguimiento (a*OR* = 6.8, IC del 95%: 1.4 – 33.7, *P* = 0.02), y la recarga de fármacos en la fecha exacta de la cita (a*OR* = 13.4, IC del 95%: 1.9 – 93.0, *P* = 0.01), se asociaron independientemente con la terminación del tratamiento.

Conclusiones: El nivel de la terminación del tratamiento fue más alto que el promedio a nivel nacional, con determinantes asociados al servicio identificados como críticos para asegurar la terminación del tratamiento. Estos datos proporcionan evidencia adicional para la necesidad de proveer apoyo al asesoramiento actualmente en curso hacia los pacientes de tuberculosis. El mejorar las oportunidades para el asesoramiento hacia pacientes de tuberculosis debe, por ende, ser rigurosamente promovido como un modo de aumentar la terminación del tratamiento de áreas rurales.

Translated from English version into Spanish by Laura C Vicente Rodriguez
